# Quercetin alleviates chronic unpredictable mild stress‐induced depression‐like behavior by inhibiting NMDAR1 with α2δ‐1 in rats

**DOI:** 10.1111/cns.14724

**Published:** 2024-04-14

**Authors:** Mingyan Wang, Xin Wei, Yugai Jia, Chaonan Wang, Xinliu Wang, Xin Zhang, Depei Li, Yuanyuan Wang, Yonggang Gao

**Affiliations:** ^1^ College of Integrative Chinese and Western Medicine Hebei University of Chinese Medicine Shijiazhuang China; ^2^ College of Basic Medical Sciences Hebei University of Chinese Medicine Shijiazhuang China; ^3^ Department of Medicine University of Missouri Columbia Missouri USA; ^4^ Hebei International Cooperation Center for Ion channel Function and Innovative Traditional Chinese Medicine Shijiazhuang China; ^5^ Hebei Key Laboratory of Chinese Medicine Research on Cardio‐Cerebrovascular Disease Shijiazhuang China

**Keywords:** CUMS, depression, HPA axis, NMDAR1, quercetin, α2δ‐1

## Abstract

**Background:**

Depression is a serious mental disorder and the most prevalent cause of disability and suicide worldwide. Chronic unpredictable mild stress (CUMS) can lead to a significant acceleration of depression development. Quercetin (Que) is a flavonoid compound with a wide range of pharmacological effects. Recent studies have shown that quercetin can improve CUMS‐induced depression‐like behavior, but the mechanism of its improvement is still unclear. α2δ‐1 is a regulatory subunit of voltage‐gated calcium channel, which can interact with N‐methyl‐D‐aspartate receptor (NMDAR) to form a complex.

**Objective:**

In this study, we found that Que could inhibit the increase of α2δ‐1 and NMDAR expression in rat hypothalamus induced by CUMS. In pain, chronic hypertension and other studies have shown that α2δ‐1 interacts with the NMDAR to form a complex, which subsequently affects the expression level of NMDAR. Consequently, the present study aimed to investigate the antidepressant effect of Que in vivo and in vitro and to explore its mechanism of action in terms of the interaction between α2δ‐1 and NMDAR.

**Methods:**

Rats were randomly exposed to two stressors every day for 4 weeks to establish a CUMS rat model, then sucrose preference test (SPT), forced swimming test (FST), tail suspension test (TST), and open field test (OFT) were performed to detect the behavior of CUMS rats, so as to evaluate whether the CUMS rat model was successfully established and the improvement effect of Que on CUMS‐induced depression‐like behavior in rats. Experimental techniques such as serum enzyme‐linked immunosorbent assay (ELISA), immunofluorescence, Western blot, and co‐immunoprecipitation, as well as in vitro experiments, were used to investigate the mechanisms by which Que exerts its antidepressant effects.

**Results:**

Behavioral and ELISA test results showed that Que could produce a reduction in the excitability of the hypothalamic–pituitary–adrenal (HPA) axis in CUMS rats and lead to significant improvements in their depressive behavior. Western blot, immunofluorescence, and co‐immunoprecipitation experiments showed that Que produced a decrease in NMDAR1 and α2δ‐1 expression levels and interfered with α2δ‐1 and NMDAR1 binding. In addition, the neural regulation mechanism of Que on antidepressant effect in PC12 cells knocked out α2δ‐1 gene was further verified. Cellular experiments demonstrated that Que led to a reversal of up‐regulation of NMDAR1 and α2δ‐1 expression levels in corticosterone‐injured PC12 cells, while Que had no effects on NMDAR1 expression in PC12 cells with the α2δ‐1 gene knockout.

**Conclusions:**

Que has a good antidepressant effect and can significantly improve the depression‐like behavior caused by CUMS. It exerts antidepressant effects by inhibiting the expression level of α2δ‐1, interfering with the interaction between α2δ‐1 and NMDAR, and then reducing the excitability of the HPA axis.

## Graphical abstract

Quercetin exerts antidepressant neuromodulation by inhibiting α2δ‐1, interfering with the interaction between α2δ‐1 and NMDAR, and reducing the expression of NMDAR.

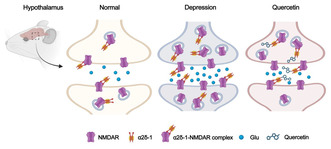

## 1 INTRODUCTION

Depression is a complex neuropsychiatric disease with a wide incidence. The global incidence of depression is reported to be as high as 4.4%.^1^ It is estimated that by 2030, depression will be the second leading cause of death worldwide, and this trend is increasing every year.^2^ Chronic unpredictable mild stress (CUMS) is one of the most important causes of depression. Long‐term exposure to the continuous bombardment of unpredictable micro‐stressors has led to large numbers of behavioral changes, the most prominent of which include the core clinical symptoms of depression with anhedonia.^3^ Many studies have shown that CUMS leads to an increase in the excitability of the hypothalamic–pituitary–adrenal (HPA) axis.^4^, ^5^, ^6^, ^7^ The HPA axis is an important neuroendocrine pathway in stress‐induced depression.^8^ Corticotropin‐releasing hormone (CRH) neurons in the hypothalamic paraventricular nucleus (PVN) are over‐activated under stress, thereby releasing a large amount of CRH, promoting the release of ACTH from the pituitary gland, increasing the expression of corticosterone (CORT), leading to HPA axis excitation and depression‐like behavior.^9^

The increased activity of PVN‐CRH neurons induced by chronic stress is mainly driven by the neural plasticity of glutamatergic afferent fibers. Glutamate (Glu) is a major excitatory neurotransmitter in the central nervous system, involved in regulating the activity of the HPA axis.^10^ Glu exerts physiological effects by acting on corresponding receptors. The N‐methyl‐D aspartate receptor (NMDAR) is the main subtype of the Glu receptor and plays an important role in synaptic plasticity, learning, memory, and emotions.^11^, ^12^ NMDAR consists of three subunits: (1) NMDAR1, (2) NMDAR2 (2A–2D), and (3) NMDAR3 (3A and 3B). The NMDAR1 subunit is a basic functional subunit and is widely expressed in the central nervous system (CNS). It is necessary for many neuronal and non‐neuronal cell functions, including plasticity, survival, and differentiation.^13^, ^14^ NMDAR controls a variety of brain functions and is related to the pathogenesis of mental disorders.^15^ Studies have shown that the presynaptic membrane NMDAR is involved in the regulation of the CNS via mediation of Glu input in addition to playing a key role in depression induced by CUMS.^16^

Generally known as a voltage‐activated Ca^2+^ channel (VACC) subunit, α2δ‐1 (encoded by CACNA2D1), is the binding site for gabapentinoids.^17^, ^18^, ^19^ α2δ‐1 is highly expressed in the hypothalamus, particularly in the PVN.^20^ α2δ‐1 can interact with NMDAR to form the complex α2δ‐1–NMDAR. α2δ‐1 expression increases in the spinal cord of rats with nerve ligation, which leads to an increase in the NMDAR current. In rats overexpressed with lentivirus CACNA2D1, the presynaptic and postsynaptic NMDAR activity of spinal dorsal horn neurons was found to be enhanced. In addition, the dynamic distribution of NMDAR in synapses was increased, which led to pain hypersensitivity. In contrast, knocking out or interfering with CACNA2D1 returned synaptic NMDAR activity with increased nerve damage to normal levels. Multiple studies have shown that the α2δ‐1–NMDAR complex mediates neuropathic pain, drug tolerance, and pain hypersensitivity induced by chemical drugs or nerve damage and is a novel analgesic target. Furthermore, it was found that depression and pain share biological pathways and neurotransmitters.^21^, ^22^, ^23^, ^24^ Moreover, the α2δ‐1–NMDAR complex was also found to be present in the neurogenic PVN of hypertensive rats and in the brain of stroke‐induced cerebral ischemia rats, suggesting that this membrane protein complex also mediates the occurrence of this series of pathological states. However, the actions of the α2δ‐1–NMDAR complex have rarely been reported in depression.

Quercetin (Que) is a natural flavonoid widely found in nature and is widely distributed in many natural plants, such as fruits and vegetables in addition to medicinal plants.^25^ Que plays a key role in the central nervous system with neuroprotective, anti‐inflammatory, and anti‐oxidative stress effects.^26^ Que also offers good antidepressant effects.^27^, ^28^, ^29^ However, only a few reports on the neuroregulatory mechanisms by which it exerts antidepressant effects, especially on the role of α2δ‐1‐NMDAR complex, have been published. Therefore, this study is based on a CUMS‐induced depression animal model to observe the antidepressant mechanism of Que on the α2δ‐1–NMDAR complex.

## 2 MATERIALS AND METHODS

### 2.1 Animals

Four‐week‐old specific pathogen‐free (SPF)‐grade Sprague Dawley (SD) male rats (140–160 g; *n* = 48) were purchased from Liaoning Changsheng Biotechnology Co., Ltd (License No. SCXK [Liao] 2020‐0001). Animal experiments were carried out strictly in accordance with the provisions and general recommendations of the Regulations on the Management of Experimental Animals in China and were approved by the Animal Ethics Committee of Hebei University of Chinese Medicine (Ethical No.: DWLL202203130). Rats were housed in clean polypropylene cages at 25 ± 2°C with a relative humidity of 25%~35% and a 12‐h day–night cycle. During the breeding period, rats were provided with standard pellet feed and ad libitum access to water.

### 2.2 Drugs and reagents

Que was purchased from Rhawn Biotechnology Co., Ltd (Shanghai, China). Fluoxetine (Flu), Corticosterone (CORT), and Gabapentin (GBP) were purchased from Shanghai Aladdin Biochemical Technology Co., Ltd (Shanghai, China). Que was suspended in 1% sodium carboxymethyl cellulose solution for in vivo experiments and dissolved in 0.1% dimethyl sulfoxide (DMSO) for in vitro experiments. Fluoxetine was dissolved in 0.9% normal saline and used as a positive control for commonly used antidepressant selective serotonin reuptake inhibitors for in vivo experiments. CORT was dissolved in DMSO, and GBP was dissolved in 0.9% normal saline for in vitro experiments. All drugs were freshly prepared before administration.

ACTH and CORT kits were purchased from Wuhan Fine Biotechnology Co., Ltd (Wuhan, China). The bicinchoninic acid (BCA) kit was purchased from Nanjing Jiancheng Bioengineering Institute (Nanjing, China). Co‐Immunoprecipitation kit was purchased from Beyotime Biotechnology Co., Ltd (Shanghai, China). RIPA lysate was purchased from Beijing Solarbio Technology Co., Ltd (Beijing, China). NMDAR1 and α2δ‐1 monoclonal antibodies were purchased from Abcam Technology Co., Ltd (Shanghai, China). Brain‐derived neurotrophic factor (BDNF), beta‐actin polyclonal antibody, secondary antibodies, goat anti‐rabbit IgG, and goat anti‐mouse IgG, were purchased from Affinity Biosciences (Jiangsu, China) for Western blotting assays. The polyclonal NMDAR1 and α2δ‐1 antibodies for immunofluorescence assay were purchased from Affinity Biosciences Co., Ltd (Jiangsu, China). The polyclonal NMDAR1 antibodies for co‐immunoprecipitation assays were purchased from Thermo Fisher Scientific Co., Ltd (Shanghai, China), and the polyclonal α2δ‐1 antibodies were purchased from Novus Biologicals Co., Ltd (Shanghai, China).

### 2.3 CUMS procedure

**FIGURE 1:**
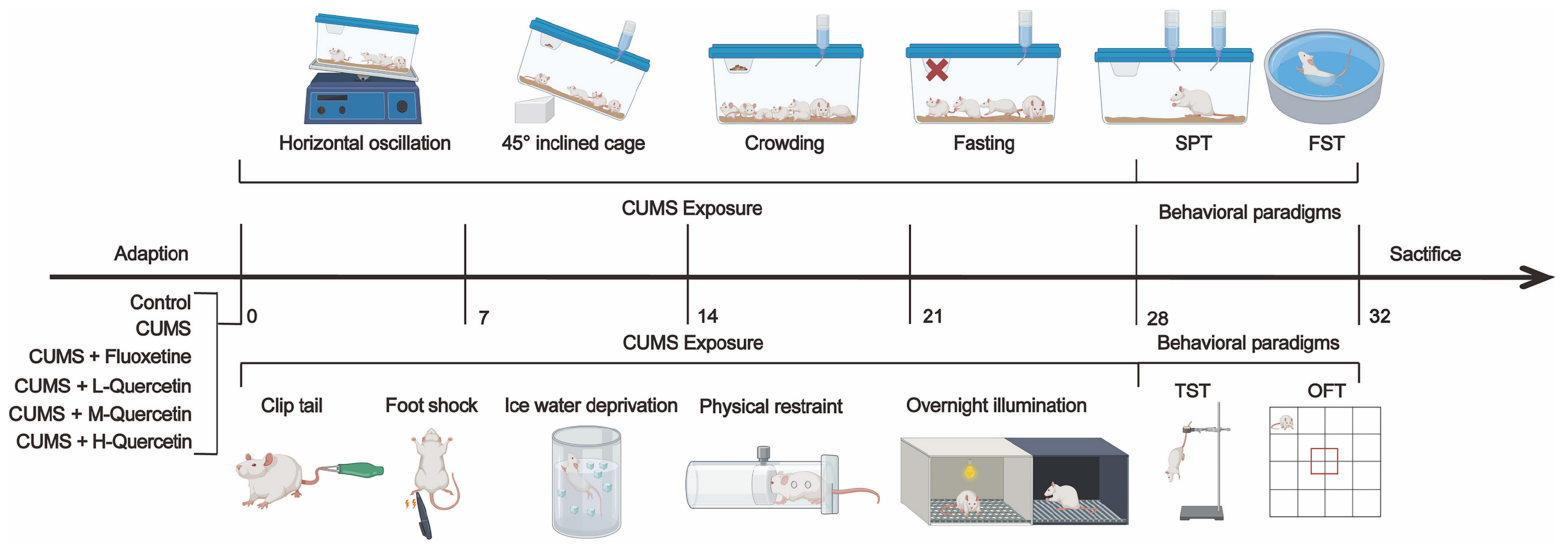
Schematic of the experimental mode.

After a week of adaptive feeding, the rats were randomly divided into six groups with eight rats in each group: (1) Control, (2) CUMS, (3) CUMS + Flu (2 mg/kg), (4) CUMS + L‐Que (low dose: 20 mg/kg), (5) CUMS + M‐Que (middle dose: 40 mg/kg), and (6) CUMS + H‐Que (high dose: 60 mg/kg). The doses of Que and Flu were determined based on previous studies and appropriate modifications.^30^, ^31^ The drugs were administered by gavage daily for 4 weeks. The control group was fed normally without any stimulation. The other groups were prepared CUMS depression model based on previous studies.^32^ Specific stressors included nine processes: (1) physical restraint (2 h), (2) crowding (12 h), (3) inclining cage at 45° (12 h), (4) swimming in ice water (6 min), (5) clipping tail (1 min), (6) foot shocks (15 times), (7) fasting (12 h), (8) horizontal oscillation (30 min), and (9) overnight illumination. Except for the control group, rats in each group were randomly given two kinds of stress‐related stimuli every day, and the same stimulation was not repeated for 3 days. The rats in each group received the same stimulation on the same day for 4 weeks (see Table S1). Behavioral experiments, including the sucrose preference test (SPT), forced swimming test (FST), open field test (OFT), and tail suspension test (TST), were performed after 4 weeks. The experimental schedule is shown in Figure 1.

### 2.4 Sucrose preference test (SPT)

SPT takes place on the day 29. Before the experiment, the rats in each group fasted for 12 h and were then given a bottle of 1% sucrose solution and a bottle of the same amount of pure water (100 mL each). The intake of sugar water was measured 2 h after administration of solutions. To avoid positional adaptation of rats, the positions of sucrose solution and pure water bottle were changed after 1 h.^33^ The sucrose preference index was calculated using the formula shown below:

$$\text{Sucrose preference}\left(\%\right)=\frac{V\left(\text{sucrose consumption}\right)}{V\left(\text{sucrose consumption}+\text{water consumption}\right)}\times 100\%$$

### 2.5 Forced swimming test (FST)

The FST was conducted on day 30. The laboratory environment was quiet throughout the experiment. The rats were placed in a container (46 cm high, 15 cm diameter) filled with 40 cm water (25 ± 2°C). The experiment lasted for 6 min, and the immobility time of the rats was recorded by two qualified observers who were blinded to the treatment. The immobility time was recorded 4 min after the rats entered the water. As long as the rat floated passively in the water and only made a slight movement to keep its head above the waterline, it was considered to be stationary. We changed the water between each test.^34^

### 2.6 Tail suspension test (TST)

The TST was performed on day 31. The rat was suspended on the tail clip 1/3 of the distance from the tip of the tail using a non‐stick adhesive, and the head was 10 cm away from the bottom of the tail clip. The camera recorded the whole process of the rats from struggling to ceasing to struggle and then to immobility within 6 min. The immobility time within 4 min after 6 min of the experiment was recorded by two observers who turned a blind eye to treatment.^35^

### 2.7 Open field test (OFT)

The OFT was carried out on day 32. In an 80 × 80 × 40 cm open box device, the bottom of the box was divided into 16 equal squares and the rats were quickly placed in the center of the box after which recording and timing began. The trajectory, total distance, and total number of times of each rat's movement through the central grid were calculated. After 5 min, the camera was stopped and the inner wall and bottom of the box were cleaned with 75% alcohol after which the box was dried completely to avoid residual urine and odor affecting the next result.^36^

### 2.8 Enzyme‐linked immunosorbent assay (ELISA)

After the behavioral experiment, the rats were fasted for 24 h and then anesthetized with pentobarbital (1%, 50 mg/kg). Blood was collected through the abdominal aorta, and serum was centrifuged for reserve. The baseline levels of serum adrenocorticotropic hormone (ACTH) and CORT were detected using an enzyme‐linked immunosorbent assay (ELISA) kit according to the instructions.

### 2.9 Immunofluorescent assays

The paraffin sections were dewaxed after which the tissue sections were exposed to antigens and then blocked dropwise with bovine serum antigen for 30 min. The primary antibody (NMDAR1 or α2δ‐1) was then incubated with the tissue sections incubation at 4°C in a flat, wet box overnight. After overnight incubation, the secondary antibody (goat anti‐Rabbit IgG) was added to the sections at room temperature in the dark for 50 min after which 4′6‐diamidino‐2‐phenylindole (DAPI) staining solution was added and incubated with the sections at room temperature in the dark for 10 min. Autofluorescence quencher was added to the circle for 5 min and rinsed under running water for 10 min. Finally, the acquired images were observed under a fluorescence microscope.

### 2.10 Western blot assay

Twenty milligrams of hypothalamus tissue were homogenized in 400 μL lysis buffer (50 mmol/L Tris–Cl, 150 mmol/L NaCl, 0.02% NaN_2_, 100 μg/mL phenylmethylsulfonyl fluoride, 1 μg/mL aprotinin, and 1% Triton X‐100) in the presence of a protease inhibitor and phosphatase inhibitor on ice for 45 min. Lysates were centrifuged at 4°C, 12,000 g/min for 10 min, and the supernatant was carefully collected. The cell samples were prepared by washing the adherent cells on a six‐well plate with phosphate‐buffered saline (PBS) and scraping them off with a cell scraper. The cell samples were centrifuged at 4°C and 7000 g/min for 5 min to discard the supernatant. The lysate was added for ultrasonic centrifugation and lysed in ice for 3 min. Lysates were centrifuged at 4°C and 12,000 g/min for 15 min to obtain the supernatant. The protein concentrations were determined using the BCA protein assay kit. The lysed samples were separated using sodium dodecyl sulfate–polyacrylamide gel electrophoresis (SDS‐PAGE) and then transferred to nitrocellulose membranes. After blocking with 5% skim milk for 2 h at room temperature, the membranes were incubated with specific primary antibodies at 4°C overnight. The nitrocellulose membranes were then incubated with horseradish peroxidase (HRP)‐conjugated secondary antibodies for 90 min, and protein bands were visualized via enhanced chemiluminescence. The gray intensity of the protein bands was analyzed using ImageJ software (NIH, Bethesda, MD).

### 2.11 Co‐immunoprecipitation

Protein A + G magnetic bead suspension was combined with mouse NMDAR1 antibody or α2δ‐1 antibody and incubated at 4°C overnight. Protein A + G beads are bound to IgG as a control. All samples were washed three times with immunoprecipitation buffer and then detected using western blotting. The amount of the α2δ‐1 protein was normalized to that of NMDAR1 on the same gel or vice versa.

### 2.12 Cell culture and construction of α2δ‐1 gene knockout cells

Normal rat adrenal pheochromocytoma (PC12) cells were used for the experiments. The Rat Cacna2d1 gene of PC12 cell was knocked out using the electroporation‐mediated clustered regularly interspaced short palindromic repeats (CRISPR)/Cas9 gene editing technology. After electroporation, monoclonal clones were selected and verified by polymerase chain reaction (PCR) and sequencing. Homozygous cells with Rat Cacna2d1 gene knockout, namely 1D10 cells, were successfully obtained (see Table S2). The cell culture system consisted of Dulbecco's modified Eagles medium (DMEM) with 20% fetal bovine serum (FBS) and 1% penicillin. The culture conditions were 37°C, 5% CO_2_ incubator, and the frequency of passaging was 2–3 days.

### 2.13 Cell modeling and treatment

Wild PC12 (WT) cells were used for in vitro experiments to establish a cellular depression model by administering CORT‐induced damage, which can mimic the nerve damage caused by excessive secretion of CORT caused by the HPA axis hyperactivity during depression. WT cells were divided into the control, CORT, and Que administration groups, and the logarithmic growth phase cells were taken and inoculated in 6‐well plates. After the cells were stable, the CORT group was treated with CORT for 24 h, and the Que group was given both CORT and Que treatment for 24 h, and the subsequent experiments were performed.

### 2.14 CCK‐8 experiments

WT and 1D10 cells were treated with different concentrations of CORT (100–800 in 100 μmol/L increments) for 24 h. Optical density (OD) values were detected, and cell survival rates were calculated, and the concentration with the closest inhibition rate of 50% was taken as the optimal modeling concentration for cells. The cells were then divided into control (untreated), CORT (CORT‐treated), and Que (CORT + Que, Que concentrations were 6.25, 12.5, 25, 50, 100, and 200 μmol/L, respectively) groups. After 24 h of different treatments, the cell survival rate was calculated, and the concentration with the most significant difference between the Que and CORT groups was used as the optimal concentration for subsequent experiments. The optimal concentration of gabapentin (GBP) for WT cells was selected by the same method.

### 2.15 Statistical analysis

SPSS v 22.0 and Origin 2021 software were used to establish the database and perform the statistical analyses. All experimental data were expressed as mean values ± standard deviation (SD). The data were tested for normality using the Shapiro–Wilk test, and all the data were in accordance with the normality test. After meeting the normal distribution, the data were tested for variance homogeneity. One‐way analysis of variance (ANOVA) followed by a Tukey test was used to compare differences between groups. When the normal distribution was not satisfied, a Wilcoxon rank sum test and Kruskal–Wallis rank sum test were used to compare the differences between groups. The significance level was set as *p* < 0.05 or *p* < 0.01.

## 3 RESULTS

### 3.1 Que alleviates depression‐like behavior in rats induced by CUMS

**FIGURE 2:**
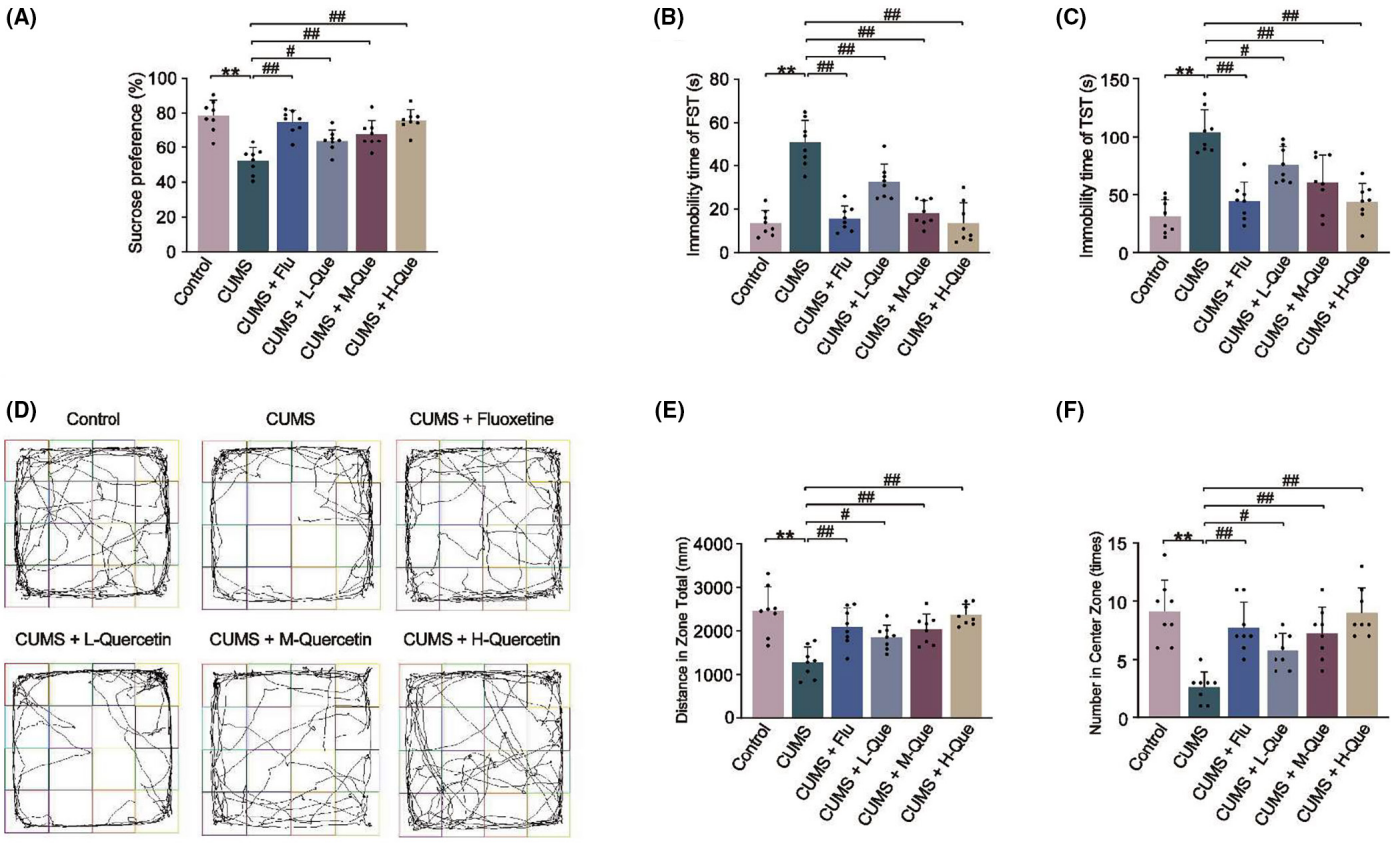
Quercetin (Que) leads to an improvement in depression, evasion, and despair in CUMS rats. (A) The sucrose preference index of SPT; (B) The immobility time of FST; (C) The immobility time of TST; (D) Trajectory diagram of each group in OFT; (E) Total movement distance of each group in OFT; (F) The number of times crossed the central grid in the OFT. The results are represented as mean ± SD (*n* = 8). The data were analyzed with One‐way ANOVA followed by Tukey's test. Compared with the control group: **p < 0.01; Compared with the CUMS group: ^#^ *p* < 0.05, ^##^ *p* < 0.01.

After modeling, we performed behavioral experiments, including SPT, FST, TST, and OFT, which were used to assess anhedonia, level of hopelessness, anxiety depressive states, and autonomic exploration capability, respectively. When compared with the control group, the sucrose preference index in the SPT, the number of times and total distance traversed through the central grid in the OFT in the CUMS group had significantly decreased, and the immobility times in the FST and TST significantly increased as shown in Figure 2. When compared with the CUMS group, the sucrose preference index, the number of times and total distance traversed through the central grid of the different intervention groups (CUMS + QL; CUMS + QM; CUMS + QH) had significantly increased, and the immobility time had significantly decreased.

### 3.2 Que inhibited CUMS‐induced HPA axis hyperactivity in rats

**FIGURE 3:**
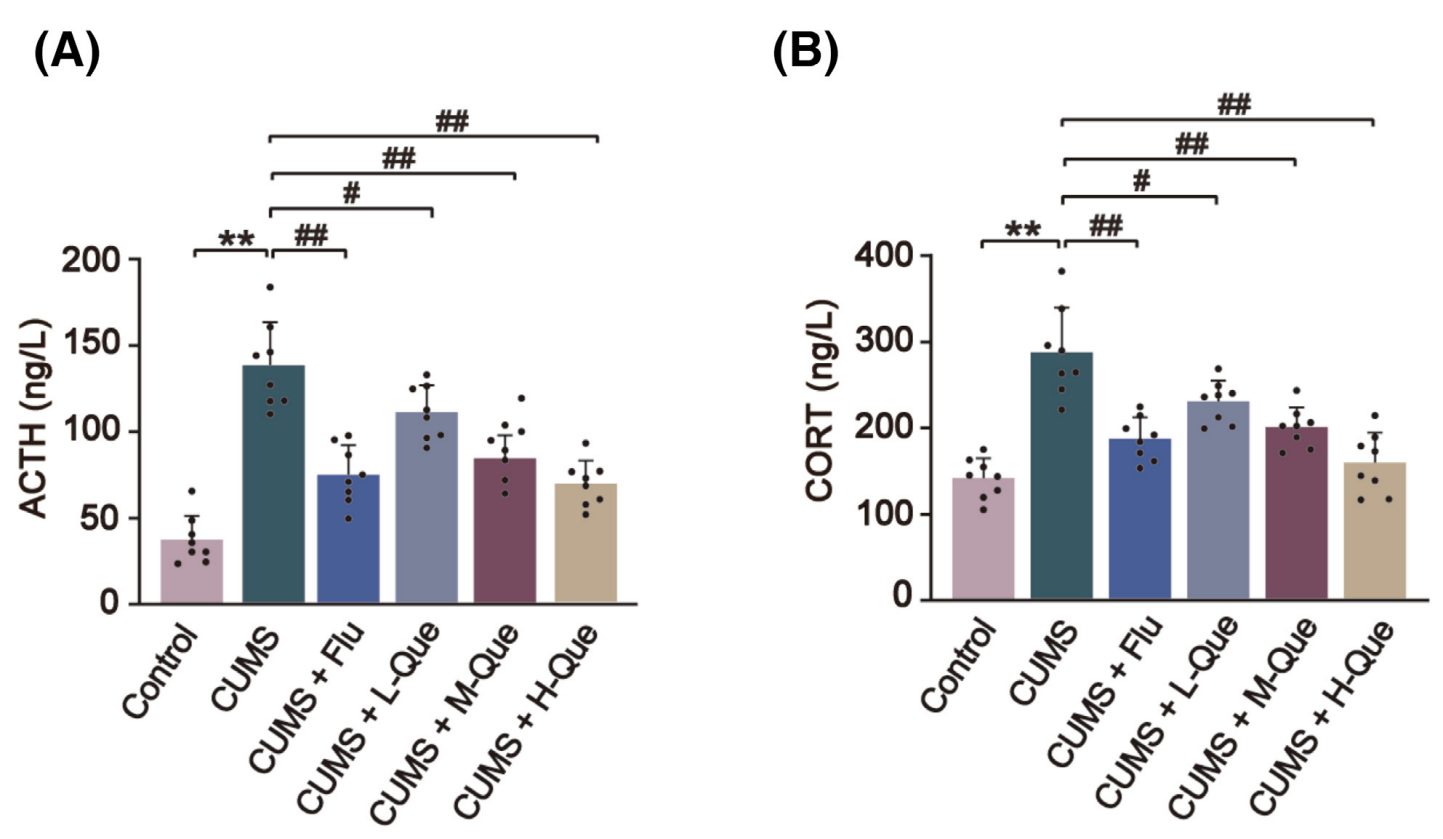
Que produced a decrease in the concentrations of serum ACTH and CORT in CUMS rats. (A) The serum baseline level of ACTH; (B) The serum baseline level of CORT in each group. The results are represented as mean ± SD (*n* = 8). The data was analyzed with One‐way ANOVA followed by Tukey's test. Compared with the control group: **p < 0.01; Compared with the CUMS group: ^#^ *p* < 0.05, ^##^ *p* < 0.01.

The baseline levels of serum ACTH and CORT in rats were detected using an ELISA. When compared with the control group, the baseline levels of serum ACTH and CORT in CUMS group had significantly increased, indicating that the HPA axis excitability in the CUMS group had significantly increased. After treatment with Que when compared with the CUMS group, the baseline levels of serum CORT and ACTH in the low, medium, and high dose Que groups were all decreased among which CUMS + H‐Que group was consistent with Flu group the most significant effects as shown in Figure 3.

### 3.3 Que produced a decrease in the expression of NMDAR1 and α2δ‐1 in the hypothalamus of CUMS rats

**FIGURE 4:**
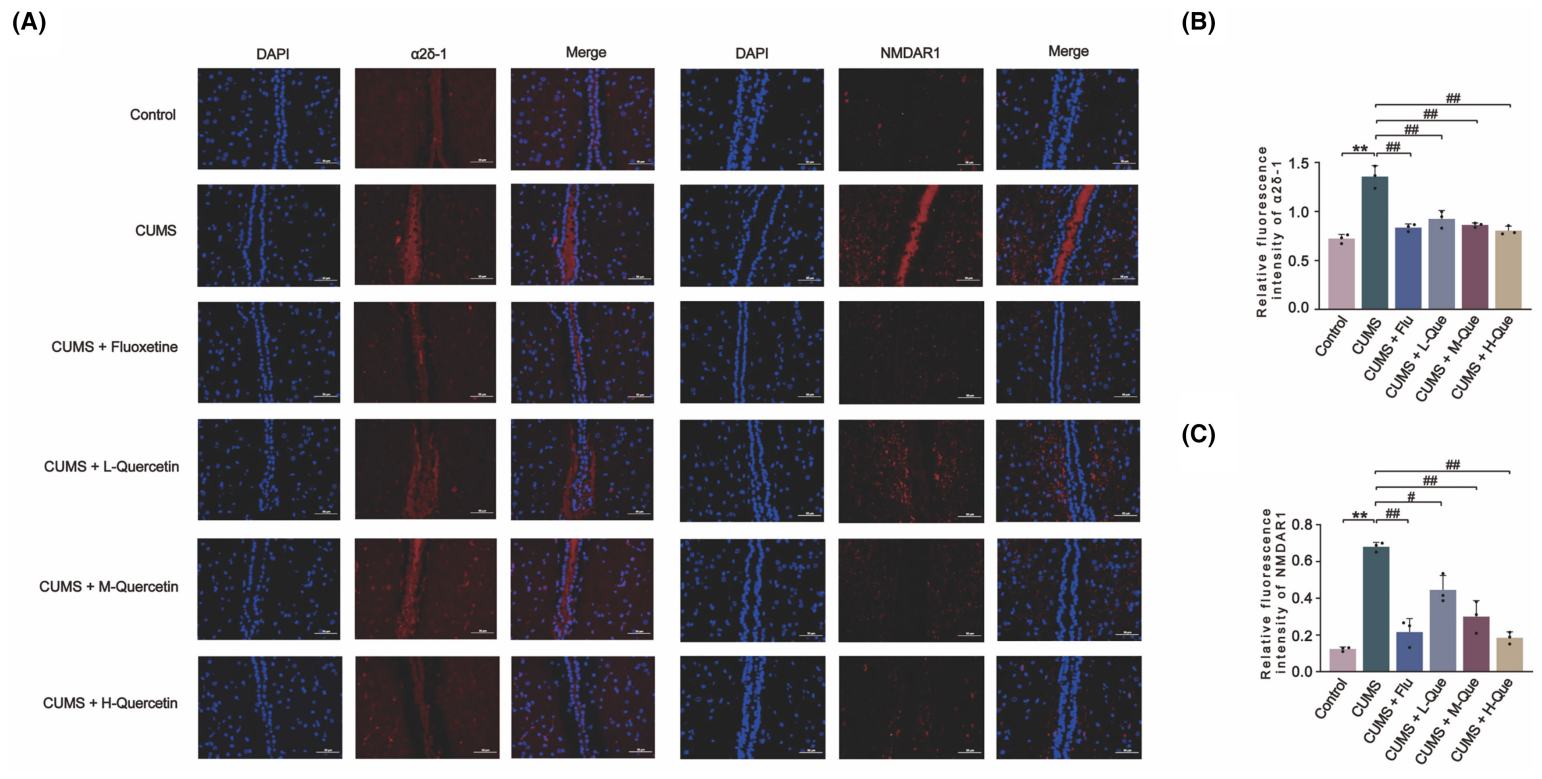
Que produced a decrease in the expression of NMDAR1 and α2δ‐1 in the hypothalamus of CUMS rats. (A) Representative images of NMDAR1 and α2δ‐1 in immunofluorescence experiments. (B) Relative fluorescence intensity of α2δ‐1. (C) Relative fluorescence intensity of NMDAR1; Mean optical density of positive reactants was calculated by Image J software. Bar = 50 μm (magnification, ×400). The results are represented as mean ± SD (*n* = 3). The data was analyzed with One‐way ANOVA followed by Tukey's test. Compared with the control group: **p < 0.01; Compared with the CUMS group: ^#^ *p* < 0.05, ^##^ *p* < 0.01.

**FIGURE 5:**
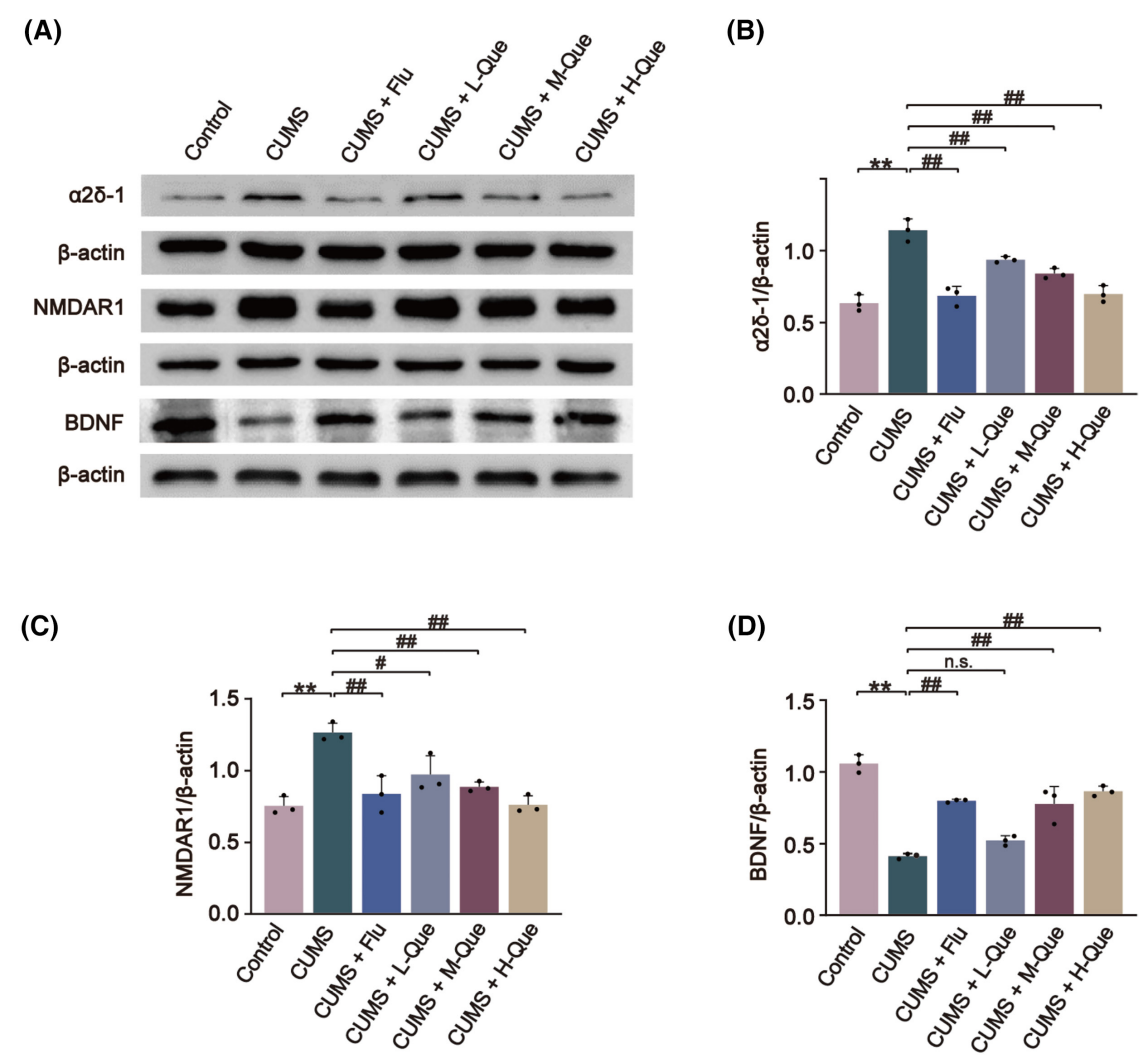
Effects of Que on the expression levels of NMDAR1, α2δ‐1, and BDNF in the hypothalamus of the CUMS rats. (A) Representative images of NMDAR1, α2δ‐1, BDNF and their respective internal references (β‐Actin) based on Western blots; (B) The ratio of protein expression level of α2δ‐1 to β‐Actin; (C) The ratio of protein expression level of NMDAR1 to β‐Actin; (D) The ratio of protein expression level of BDNF to β‐Actin. The results are represented as mean ± SD (*n* = 3). The data was analyzed with One‐way ANOVA followed by Tukey's test. Compared with the control group: **p < 0.01; Compared with the CUMS group: ^#^ *p* < 0.05, ^##^ *p* < 0.01; n.s., No significance.

The expression of NMDAR1 and α2δ‐1 in the hypothalamus of CUMS rats was detected using immunofluorescence assay and Western blotting method. When compared with the control group, the expression level of NMDAR1 and α2δ‐1 in the hypothalamus of the CUMS group had significantly increased. When compared with CUMS group, different concentrations of Que intervention led to a decrease in the expression levels of NMDAR1 and α2δ‐1. CUMS + H‐Que group had the most significant effect and resembled results in the Flu treatment group (Figures 4 and 5).

Western blotting was also used to detect the expression levels of BDNF in the hypothalamus of CUMS rats. The expression levels of BDNF protein in the CUMS group had significantly decreased when compared with the control group. After Que treatment, the expression levels of BDNF in the CUMS + M‐Que, and CUMS + H‐Que groups had increased. Furthermore, no significant difference in the expression level of BDNF protein between CUMS + L‐Que group and CUMS group (Figure 5A,D).

### 3.4 Que inhibits the interaction of α2δ‐1 with NMDAR1 in CUMS rats

**FIGURE 6:**
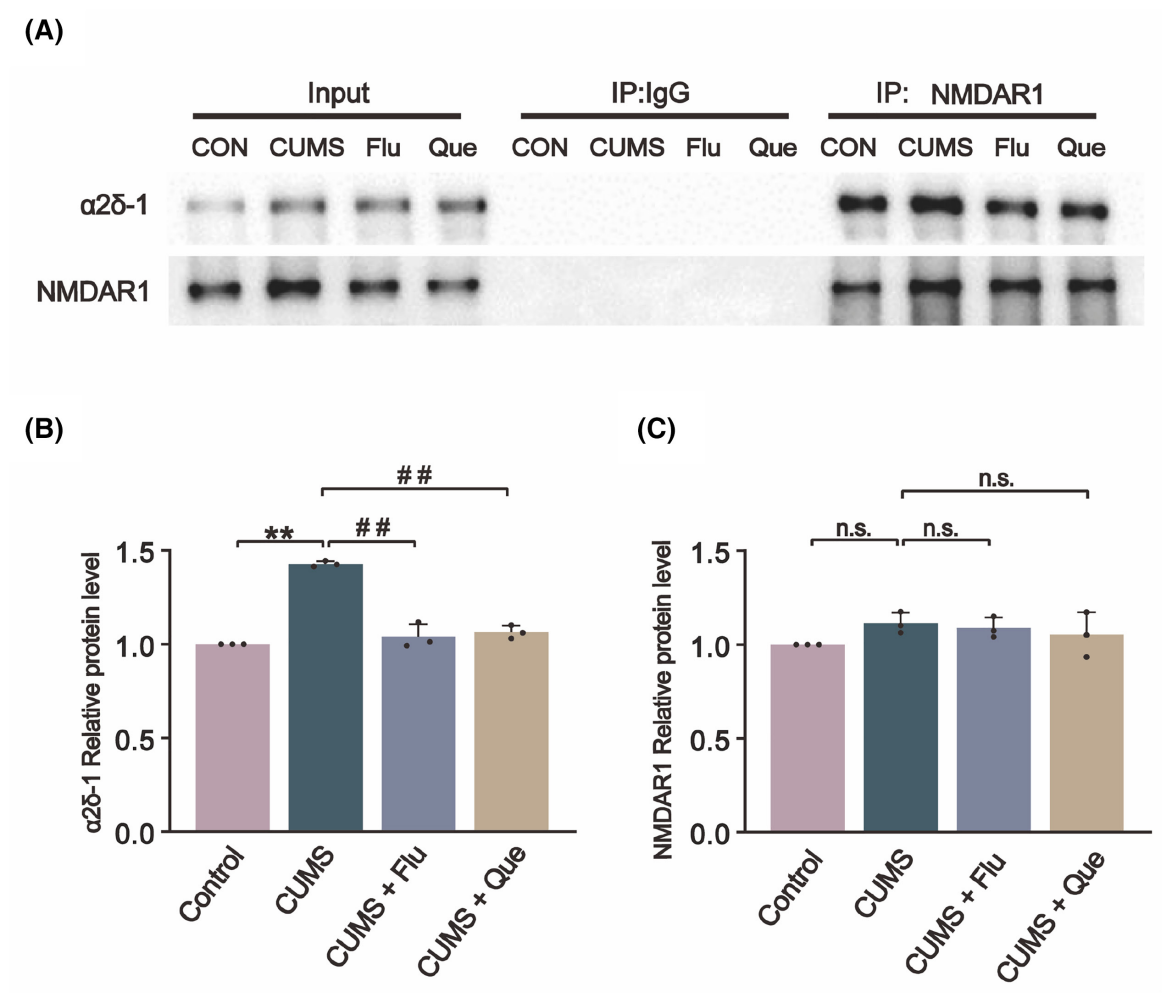
Co‐immunoprecipitation analysis shows the interaction between α2δ‐1 and NMDAR1 in total protein extracts of PVN tissues from control, CUMS, and CUMS + H‐Que rats. (A) The protein was initially immunoprecipitated (IP) with NMDAR1 antibody or IgG antibody, and then immunoblotted with α2δ‐1 antibody; (B) The amount of α2δ‐1 proteins was normalized to that of NMDAR1 on the same blot; (C) The amount of NMDAR1 proteins on the same blot; The results are represented as mean ± SD (*n* = 3). The data was analyzed with One‐way ANOVA followed by Tukey's test. Compared with the control group: **p < 0.01; Compared with the CUMS group: ^##^ *p* < 0.01; n.s., No significance.

To determine whether α2δ‐1 and NMDAR1 interact in CUMS rats, we conducted co‐immunoprecipitation analyses using total proteins extracted from the hypothalamus of control, CUMS, and CUMS + H‐Que rats. The anti‐α2δ‐1 antibody co‐precipitated with NMDAR1 in the hypothalamic tissues obtained from these rats, whereas the irrelevant IgG did not co‐precipitate with NMDAR1 (Figure 6). This finding suggests that an interaction between α2δ‐1 and NMDAR1 in CUMS rats occurred, and the level of the α2δ‐1–NMDAR1 complex was much greater in the CUMS group than in the control group. When compared with CUMS group, the α2δ‐1–NMDAR1 complex level in Que and Flu groups was significantly lower.

### 3.5 Que inhibited CORT‐induced damage in WT and 1D10 cells

The survival rates of WT and 1D10 cells were measured using the cell counting kit assay 8 (CCK‐8) method with different concentrations of CORT (100–800 μmol/L in 100 μmol/L increments). The results showed that when CORT concentration was 500 μmol/L, the survival rate of WT cells was 51.7% and that of 1D10 cells was 50.1%; thus, the concentration with the inhibition rate closest to 50% was taken as the best concentration for the model. Cells were treated with different concentrations of Que (6.25, 12.5, 25, 50, 100, or 200 μmol/L) for 24 h. OD values were measured after incubation with CCK‐8 reagent, and cell survival rates were calculated. The results showed that 100 μmol/L Que produced the best therapeutic effects on WT and 1D10 cells, and the difference was most significant when compared with the CORT group. The survival rate of WT cells was 50.47% after 40 mmol/L GBP treatment with different concentrations of α2δ‐1 inhibitor GBP (see Figure S1).

### 3.6 Que inhibits α2δ‐1 and NMDAR1 expression levels in CORT‐injured WT and 1D10 cells

**FIGURE 7:**
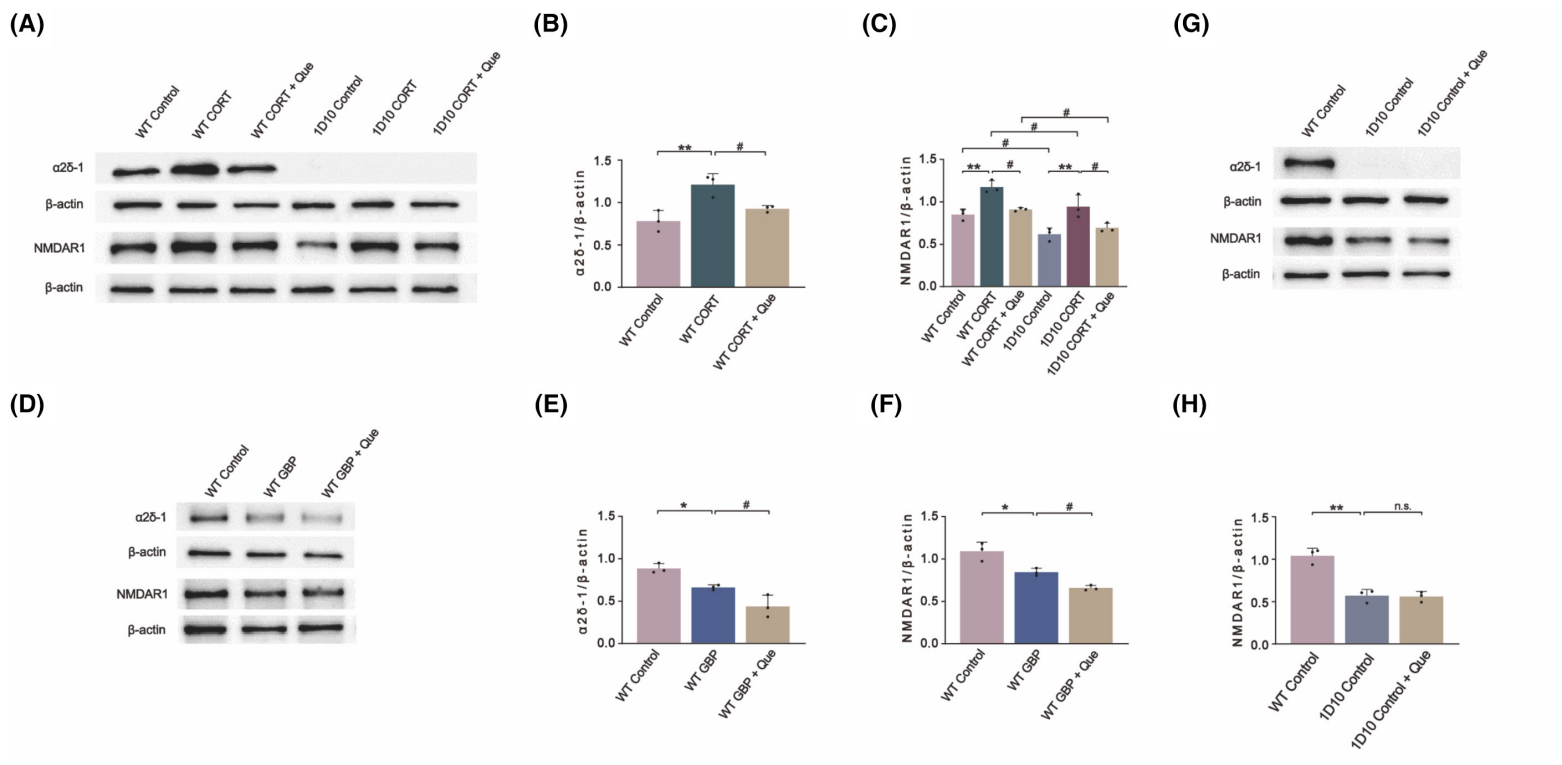
Effect of Que on the expression levels of α2δ‐1 or NMDAR1 in PC12 cells after administration of CORT, GBP treatment, and knockout of the α2δ‐1 gene. (A) The representative images of NMDAR1 and α2δ‐1 and their respective internal reference (β‐Actin) in Western blot after CORT treatment of WT cells and 1D10 cells; (B) The ratio of protein expression level of α2δ‐1 to β‐Actin; (C) The ratio of protein expression level of NMDAR1 to β‐Actin; (D) The representative images of NMDAR1 and α2δ‐1 and their respective internal reference (β‐Actin) in Western blot after GBP treatment of WT cells; (E) The ratio of protein expression level of α2δ‐1 to β‐Actin; (F) The ratio of protein expression level of NMDAR1 to β‐Actin; (G) Representative images of NMDAR1 and α2δ‐1 from protein imprints and respective internal references (β‐Actin) after intervention by Que to 1D10 cells; (H) The ratio of protein expression level of NMDAR1 to β‐Actin. The results are represented as mean ± SD (*n* = 3). The data was analyzed with One‐way ANOVA followed by Tukey's test. Compared with the control group: *p < 0.05, **p < 0.01; Compared with the CUMS group: ^#^ *p* < 0.05; n.s., No significance.

In this study, Western blotting experiments were performed with WT cells and 1D10 cells treated with CORT and Que, and the expression levels of α2δ‐1 and NMDAR1 in the depression cell model were further verified (Figure 7A–C). The expression levels of α2δ‐1 and NMDAR1 in WT cells after CORT treatment were significantly higher than those in the control group, but the expression levels of α2δ‐1 and NMDAR1 after Que treatment had decreased. The expression levels of NMDAR1 in 1D10 cells were consistent with those in WT cells. Interestingly, the expression levels of NMDAR1 in the 1D10 cells were significantly lower than those in the WT cells, indicating that the knockout of α2δ‐1 would lead to a reduction in the expression of NMDAR1, while Que had little effect (Figure 7G,H).

### 3.7 Que affects the expression of NMDAR1 by regulating the expression of α2δ‐1

To further verify the effects of Que on the expression levels of α2δ‐1 and NMDAR1, WT cells were divided into control, GBP (α2δ‐1 inhibitor), and GBP + Que groups. When compared with the control group, the expression levels of α2δ‐1 and NMDAR1 in the GBP group were significantly down‐regulated. After Que treatment, the expression levels of α2δ‐1 and NMDAR1 had further decreased when compared with the GBP group (Figure 7D–F).

## 4 DISCUSSION

In this study, Que was found to exert an antidepressant effect by down‐regulating the expression of α2δ‐1, a process that can affect the activity and expression of NMDAR1.

Currently, the clinical treatment of depression is mainly based on selective 5‐hydroxytryptamine reuptake inhibitors, and the pathway of action is relatively simple. Not only are patients prone to developing tolerance to the drug, but they also experience nausea, insomnia, headache, and other adverse reactions. With the ongoing and extensive research concerning the mechanism of Glu excitatory neurotransmitters, NMDAR inhibitors have become the focus of antidepressant drug development in recent years. Ketamine, for example, is a general anesthetic. It has a rapid and sustained antidepressant effect at low doses, but its safety and efficacy need to be further studied, and its clinical use is limited. α2δ‐1 regulates the expression of NMDAR and may provide a new target for the treatment of depression.

Que is a flavonoid compound that is rich in many natural plants and has a wide range of pharmacological effects and nutritional value. Que has many biological activities and plays a key role in the central nervous system, plays a beneficial role in neuroprotection, and inhibits HPA axis excitability. In recent years, studies have shown that Que has a certain antidepressant effect,^37^, ^38^, ^39^ a finding that was further verified in this study.

Depression is a mental disorder characterized by high morbidity and mortality.^40^ Prolonged elevation of cortisol caused by chronic stress leads to maladaptive changes and the occurrence of various disease states in the body. Chronic stress includes sleep deprivation, social isolation, and trauma, all of which increase the susceptibility to depression and threaten the organism, making chronic stress one of the most critical factors in the development of depression.^41^, ^42^ CUMS can mimic the typical clinical symptoms of depression found in pleasure disorders.^43^ CUMS is currently the most frequently used, reliable, and validated rodent model of depression. Therefore, in this study, we established a CUMS model by exposing rats to a random stressor for 4 weeks^44^ after which SPT, FST, TST, and OFT behavioral experiments were used to evaluate the CUMS model and the antidepressant effects of Que. The experimental results showed that CUMS led to a significant reduction in the sucrose preference index and prolonged the immobility time of FST and TST. In addition, Que led to a reduction in the total distance of OFT movement and the number of times traversing the central grid of the rats, a finding that proved that the CUMS rat model had been successfully established. After Que treatment, the behavioral indicators of CUMS rats were reversed, indicating that Que had a significant improvement effect on CUMS‐induced depression‐like symptoms. Higher Que doses produced more significant effects.

Stress leads to structural and functional changes in several regions of the brain, among which the hypothalamus is an important brain region regulating emotions and behaviours,^45^ and the aberrant activation of the neuroendocrine system downstream of the hypothalamus, including the pituitary gland and the adrenal cortex, is generally considered to be an important component of the stress system.^46^, ^47^ ACTH and CORT are the main indicators reflecting the state of the HPA axis. The baseline levels of serum ACTH and CORT were determined in all groups. It was found that the baseline levels of expression of ACTH and CORT were significantly up‐regulated in CUMS rats and then significantly decreased after Que intervention, a finding that proves that Que can ameliorate the depression‐like behaviors of CUMS rats by producing a decrease in the excitability of the HPA axis.

NMDAR is an important target that connects stress and HPA axis hyperactivity. Glu is a major excitatory neurotransmitter in the central nervous system and is involved in regulating the activity of the HPA axis. Glu exerts physiological effects by acting on the corresponding receptors, and NMDAR is the main receptor mediating the neuroexcitatory toxicity of Glu.^48^ NMDAR plays a key role in synaptic communication, mainly through its ionophilic function of permeabilizing Ca^2+^. NMDAR is considered a classical ionotropic channel that plays a central role in neuronal communication.^12^ It has been pointed out that the action of Glu on NMDAR leads to the recurrent opening of the Ca^2+^ channel, which then causes an increase in the excitability of Glu neurons and PVN‐CRH neurons and leads to the hyperfunction of HPA axis, thus inducing depressive symptoms.^49^ The results from Western blotting and immunofluorescence experiments showed that the expression level of NMDAR1 in the hypothalamus of CUMS rats was up‐regulated, which was then significantly down‐regulated after Que treatment. NMDAR can also be involved in the regulation of BDNF activity and expression. In a study of Alzheimer's disease, it was suggested that overexpression of NMDAR could lead to increased intracellular Ca^2+^ levels, which would then lead to the excessive activation of calpain, thus affecting the activity and expression of BDNF.^50^ Studies have shown that the activity and expression of BDNF play an indispensable role in the pathogenesis of depression.^51^, ^52^ The results based on Western blotting showed that the expression levels of BDNF in the hypothalamus of CUMS rats were significantly down‐regulated, and Que could inhibit this down‐regulation. BDNF is a downstream protein of NMDAR, which further proves that CUMS will lead to an increase in the expression level of NMDAR; Que can lead to a reversal in this increase. Our subsequent experiments will conduct deeper research and verification on the downstream targets of NMDAR.

The activity and expression of NMDAR is mediated by α2δ‐1. In a study of calcineurin inhibitor‐induced hypertension, it was suggested that gabapentin or α2δ1 gene knockout could eliminate tacrolimus‐induced NMDAR hyperactivity in hypothalamic sympathetic neurons by inhibiting α2δ‐1.^53^ A previous study showed excess α2δ‐1 protein physically interacted with NMDAR and enhanced NMDAR activity.^54^ In addition, studies of persistent hypertension caused by chronic stress indicated that chronic stress leads to up‐regulation of α2δ‐1 and α2δ‐1‐NMDAR complexes and an increase in NMDAR current amplitude.^55^ The α2δ‐1–NMDAR complex is widely distributed in neuronal cells and affects a variety of neurological behaviors in animals but has not been applied to depression research. Based on the above studies, it is suggested that α2δ‐1 may be associated with chronic stress‐induced depression. Western blotting and immunofluorescence results showed that α2δ‐1 was up‐regulated in hypothalamic tissue of CUMS rats and down‐regulated after Que treatment. To verify the interaction between α2δ‐1 and NMDAR, we also conducted co‐immunoprecipitation experiments, the results of which showed that the interaction between α2δ‐1 and NMDAR1 was closely related. According to the previous experimental results, we selected the group with the high dose of Que as the treatment group and normalized the expression levels of the target proteins.^56^ The results suggested that the interaction between α2δ‐1 and NMDAR1 was enhanced in CUMS rats but became weaker after Que treatment.

We further verified the role of α2δ‐1 and NMDAR through in vitro experiments in addition to the pathway of Que's antidepressant effect. PC12 cells, the cell line of rat adrenal pheochromocytoma, have typical characteristics of neuroendocrine cells in morphology and function and are often used as ideal cell models for the study of neurodegenerative diseases.^57^ When chronically exposed to stress levels of CORT, the release of serotonin can be reduced, leading to neurodegeneration. High concentrations of CORT can induce neurotoxicity in PC12 cells.^58^ Therefore, the PC12 cell model of CORT injury can simulate nerve damage caused by hyperfunction of the HPA axis, which leads to excessive secretion of corticosterone in patients with depression and has become the most frequently used cell model for depression.^59^, ^60^ In this study, to verify the interaction of α2δ‐1 and NMDAR in addition to the mechanism of Que, two types of cells were used: (1) normal PC12 cells, namely WT cells and (2) PC12 cells with stable knockout of α2δ‐1 gene, namely 1D10 cells. Both cells were damaged by CORT and treated with Que. Western blotting results showed that α2δ‐1 gene knockout results in a decrease in intracellular NMDAR1 levels, and Que leads to a reduction the expression of α2δ‐1, thereby reducing the expression of NMDAR1, which has a good antidepressant effect. These results suggested that Que is an inhibitor of α2δ‐1 and affects the expression of NMDAR1 via regulation of the α2δ‐1 expression.

## 5 CONCLUSION

In summary, our results indicated that Que produces good anti‐depressant effects and may provide a new choice for the prevention and treatment of clinical depression. In vitro and in vivo experiments show that Que may have an antidepressant role via down‐regulation of α2δ‐1 and interfere with the interaction between α2δ‐1 and NMDAR, thus affecting the activity and expression of NMDAR, inhibiting the HPA axis hyperactivity, and leading to an increase in BDNF expression. We believe that Que may be a promising candidate drug for depression treatment, and further research will be carried out on the neural regulation mechanism behind its antidepressant effects.

## AUTHOR CONTRIBUTIONS

Mingyan Wang and Xin Wei: performed research, analyzed data, and wrote the paper; Yugai Jia, Chaonan Wang, Xinliu Wang, Depei Li, and Xin Zhang: undertook the data acquisition and analysis. Yuanyuan Wang and Yonggang Gao: designed research, comprehensive technical support, and wrote the paper. Moreover, Yonggang Gao approved the final version of the manuscript and provide financial support. All authors have read and agreed to the published version of the manuscript.

## FUNDING INFORMATION

We gratefully acknowledge the stipend of Key Project of the Joint Fund of Traditional Chinese Medicine of the Natural Science Foundation of Hebei Province (H2022423381); Natural Science Fund Project of Hebei Province (H2020423004); Central Guiding Local Science and Technology Development Fund Projects (236Z7702G); Science Research Project of Hebei Education Department (QN2022006); Excellent Young Teacher Fundamental Research of Hebei University of Chinese Medicine (YQ2020009); Ministry of Education's ‘Chunhui Plan’ Cooperative Research Project (202200159); Special fund for basic scientific research expenses of provincial colleges and universities of Hebei University of Traditional Chinese Medicine (YXTD2023003); Doctoral research Fund project of Hebei University of Traditional Chinese Medicine (BSZ2019011).

## CONFLICT OF INTEREST STATEMENT

The authors declare that they have no known competing financial interests or personal relationships that could have appeared to influence the work reported in this paper.

## Supporting information


Data S1

## Data Availability

The data sets used in the current study are applicable from the corresponding author on reasonable requests.
