# Supplementary material for: Quercetin alleviates chronic unpredictable mild stress‐induced depression‐like behavior by inhibiting NMDAR1 with α2δ‐1 in rats
Source: CNS Neurosci Ther. 2024 Apr 14;30(4):e14724. doi: 10.1111/cns.14724 (PMC11016343; doi:10.1111/cns.14724)
Supplement: Supplementary file 1 — Data S1 [file CNS-30-e14724-s001.pdf]

Quercetin alleviates chronic unpredictable mild stress-induced depression-like behavior by inhibiting NMDAR1 with α2δ-1 in rats

Mingyan Wang[1#], Xin Wei[1#], Yugai Jia[2], Chaonan Wang[2], Xinliu Wang[1], Xin Zhang[1], Depei Li[3], Yuanyuan Wang[2*], Yonggang Gao[2,4,5*]

[1] College of Integrative Chinese and Western Medicine, Hebei University of Chinese Medicine, Shijiazhuang 050200, China.

[2] College of Basic Medical Sciences, Hebei University of Chinese Medicine, Shijiazhuang 050200, China.

[3] University of Missouri, Department of Medicine, Columbia, Missouri, 65203，USA.

[4] Hebei International Cooperation Center for Ion channel Function and Innovative Traditional Chinese Medicine. Shijiazhuang 050091, China.

[5] Hebei Key Laboratory of Chinese Medicine Research on Cardio-Cerebrovascular Disease, Shijiazhuang 050091, China.

[#] These authors contributed equally to this work.

Correspondence to: Yong-gang Gao: gyg3177@163.com; Yuan-yuan Wang: wangyy0830@iccas.ac.cn

Table S1 The list of CUMS procedure details.

Day	Time	Stressor	Duration	Time	Stressor	Duration
1	8:00	Crowding	12 h	20:00	Clip tail	1 min
2	8:00	Light off	12 h	21:00	Ice water	6 min
3	8:00	Physical restraint	2 h	12:00	Foot shock	15 times
4	8:00	Food deprivation	12 h	20:00	Light on	12 h
5	8:00	45°inclined cage	12 h	22:00	Clip tail	1 min
6	8:00	Physical restraint	2 h	12:00	Foot shock	15 times
7	8:00	Light off	12 h	20:00	Food deprivation	12 h
8	8:00	Crowding	12 h	21:00	Clip tail	1 min
9	8:00	Physical restraint	2 h	10:00	45°inclined cage	12 h
10	8:00	Ice water	6 min	11:00	Foot shock	15 times
11	8:00	45°inclined cage	12 h	20:00	Food deprivation	12 h
12	8:00	Clip tail	1 min	20:00	Light on	12 h
13	8:00	Ice water	6 min	11:00	Foot shock	15 times
14	8:00	Physical restraint	2 h	20:00	Food deprivation	12 h
15	8:00	Crowding	12 h	21:00	Clip tail	1 min
16	8:00	Light off	12 h	21:00	Ice water	6 min
17	8:00	Physical restraint	2 h	16:00	Foot shock	15 times
18	8:00	45°inclined cage	12 h	22:00	Clip tail	1 min
19	8:00	Crowding	12 h	20:00	Food deprivation	12 h
20	8:00	Foot shock	15 times	20:00	Light on	12 h
21	8:00	Physical restraint	2 h	20:00	45°inclined cage	12 h
22	8:00	Crowding	12 h	21:00	Clip tail	1 min
23	8:00	Ice water	6 min	11:00	Foot shock	15 times
24	8:00	Physical restraint	2 h	20:00	45°inclined cage	12 h
25	8:00	Light off	12 h	22:00	Clip tail	1 min
26	8:00	Crowding	12 h	20:00	Food deprivation	12 h
27	8:00	Ice water	6 min	20:00	45°inclined cage	12 h
28	8:00	Physical restraint	2 h	16:00	Foot shock	15 times

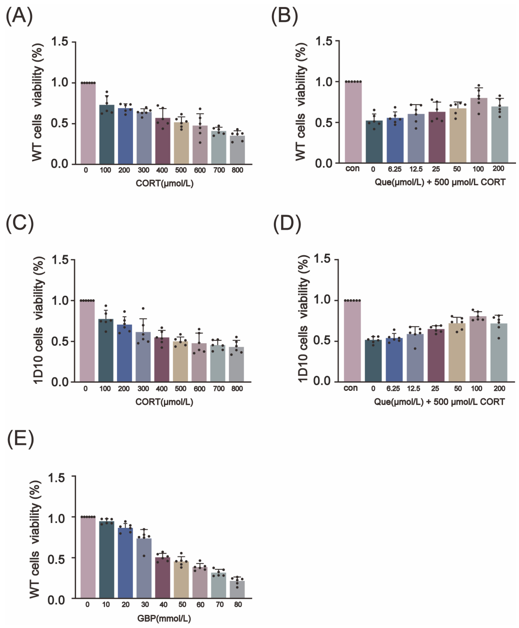

FIGURE S1 The effects of different concentrations of CORT, GBP and Que on the viability of WT cells and 1D10 cells. (A): The effect of different concentrations of CORT on the viability of WT cells; **(B):** The effect of different concentrations of Que on the survival rate of WT cells treated with 500 μmol/L CORT; **(C):** The effect of different concentrations of CORT on the viability of 1D10 cells; **(D):** The effect of different concentrations of Que on the survival rate of 1D10 cells treated with 500 μmol/L CORT; **(E):** The effect of different concentrations of GBP on the viability of WT cells.

Table S2 Gene knockout cell-related identification information

**Identification information**	
**CRISPR/Cas9 information:**	
gRNA-B1:	ACACTTGCCACAGCAATGTA-GGG
**Primers for Region 1:**	
upstream primer (SKO549-F1)	ATGTCCATCAGTCTGCCATCG
downstream primer (SKO549-R1):	GGGACAGCCACTGGAGAATTT
**Sequencing primers:**	
upstream primer (SKO549-F1):	ATGTCCATCAGTCTGCCATCG
downstream primer (SKO549-R2):	GAGAATTTAGCAGTGCCCACAT
